# Extensive Flexural Basal Cell Carcinomas Revealing Gorlin-Goltz Syndrome With Marked Histopathologic Diversity: A Case Report

**DOI:** 10.7759/cureus.109102

**Published:** 2026-05-18

**Authors:** Kawtar El Fid, Zakia Douhi, Chaimae Bouhamdi, Layla Tahiri Elousrouti, Fatima Zahra Mernissi

**Affiliations:** 1 Dermatology, Hassan II University Hospital, Faculty of Medicine, Pharmacy and Dental Medicine, Sidi Mohamed Ben Abdellah University, Fez, MAR; 2 Human Pathology, Hassan II University Hospital, Faculty of Medicine, Pharmacy and Dental Medicine, Sidi Mohamed Ben Abdellah University, Fez, MAR

**Keywords:** basal-cell carcinoma, fibroepithelioma of pinkus, gorlin-goltz syndrome, intertriginous distribution, odontogenic keratocysts

## Abstract

Gorlin-Goltz syndrome is a rare autosomal dominant disorder characterized by multiple basal cell carcinomas (BCCs), odontogenic keratocysts, and skeletal anomalies. BCCs in this syndrome typically involve photo-exposed areas, whereas predominant flexural and intertriginous involvement remains exceptionally rare. We report the case of a 42-year-old Moroccan man with Fitzpatrick phototype IV who presented with a more than 30-year history of progressively increasing pigmented and erythematous lesions. Clinical examination revealed more than 100 papules, plaques, and nodules involving the face, axillary folds, inguinal folds, and umbilical region. Some inguinal lesions became ulcerated and symptomatic, with pain and bleeding. Dermoscopy showed characteristic features of BCC, including arborizing vessels, blue-gray ovoid nests, and globules, leading to multiple biopsies that confirmed the diagnosis. Histopathological examination identified three distinct subtypes, nodular, adenoid-cystic, and fibroepithelioma of Pinkus, highlighting marked histopathological heterogeneity. A panoramic jaw radiograph demonstrated multiple odontogenic keratocysts, while chest radiography revealed bifid ribs, fulfilling major diagnostic criteria for Gorlin-Goltz syndrome. Genetic testing was not performed because of limited availability. The patient underwent surgical excision of the most symptomatic lesions. Targeted therapy with the Hedgehog pathway inhibitor vismodegib was considered but was not accessible in our setting. This case highlights a rare flexural-predominant phenotype of Gorlin-Goltz syndrome and emphasizes the importance of recognizing atypical non-sun-exposed presentations to avoid diagnostic delay and ensure appropriate multidisciplinary management.

## Introduction

Gorlin-Goltz syndrome (GGS), also known as nevoid basal cell carcinoma syndrome (NBCCS), is a rare autosomal dominant disorder most commonly caused by pathogenic variants in the *PTCH1* gene, located on chromosome 9q22.3, which is a key regulator of the Hedgehog signaling pathway [1,2]. The syndrome exhibits variable expressivity and multisystem involvement, predisposing affected individuals to basal cell carcinomas (BCCs), odontogenic keratocysts, skeletal anomalies, and other neoplasms, such as medulloblastomas or ovarian fibromas [3].

Classically, patients develop numerous BCCs predominantly in photo-exposed areas, particularly the face, neck, and upper trunk, usually beginning in adolescence or early adulthood. Although lesions may occasionally occur in sun-protected sites, predominant involvement of flexural and intertriginous regions remains exceptionally rare. Herein, we report an unusual presentation of Gorlin-Goltz syndrome characterized by extensive, bilateral, and symmetrical BCC involvement of the axillary folds, inguinal folds, and umbilical region, associated with marked histopathological heterogeneity, including nodular, adenoid-cystic, and fibroepithelioma of Pinkus subtypes occurring simultaneously in the same patient.

## Case presentation

**Figure 1:**
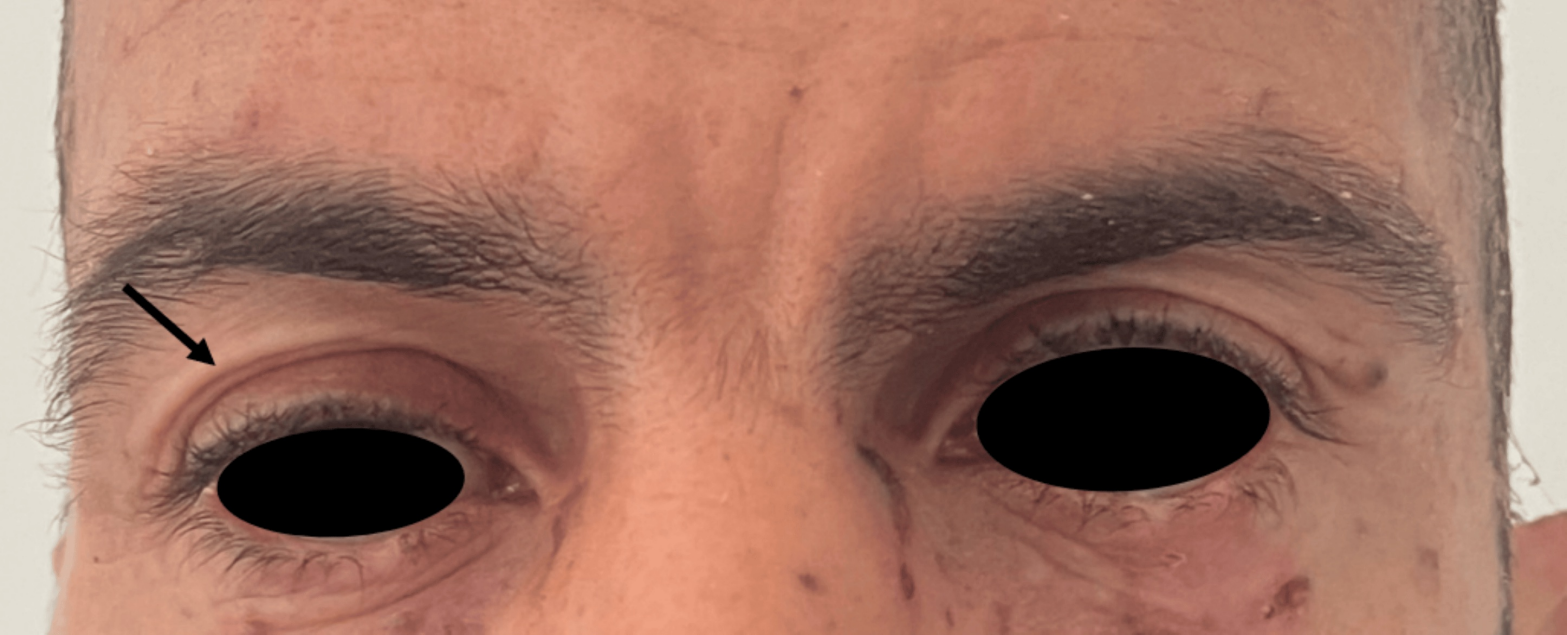
Facial appearance showing mild right upper eyelid ptosis (black arrow), without hypertelorism.

A 42-year-old Moroccan man presented with a long-standing history of progressively increasing pigmented and erythematous lesions evolving over more than 30 years. There was no known family history of similar cutaneous lesions, jaw cysts, or congenital anomalies. Facial examination of this patient with Fitzpatrick phototype IV revealed mild right upper eyelid ptosis without hypertelorism (Figure 1).

**Figure 2:**
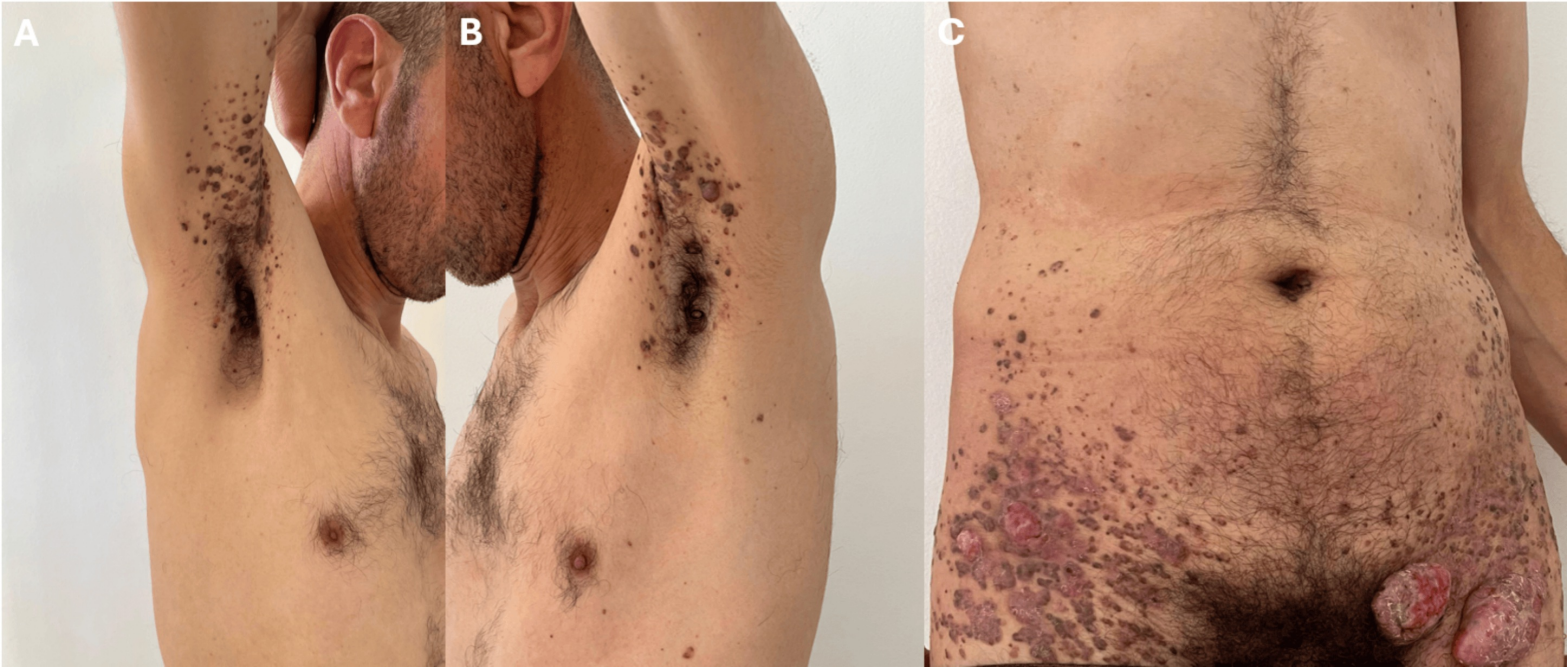
Clinical presentation of Gorlin-Goltz syndrome with multiple cutaneous lesions. (A, B) Numerous erythematous papules, plaques, and nodules distributed across the bilateral axillary folds. (C) Bilateral inguinal and periumbilical involvement; the largest lesion measures approximately 6 cm in greatest diameter.

Clinical examination of the body revealed more than 100 papules, plaques, and nodules with firm, infiltrated bases, ranging in size from a few millimeters to over 8 cm in diameter. Although a limited number of lesions involved photo-exposed areas of the face and neck, the vast majority predominantly affected flexural and intertriginous regions, including the axillae, inguinal folds, umbilical region, and flanks. Several lesions exhibited central ulceration or had coalesced into large tumoral masses, particularly in the inguinal region, leading to pain, bleeding, visible disfigurement, and local discomfort (Figure 2). Oral examination revealed poor oral hygiene associated with dental malalignment. Ophthalmologic examination was otherwise unremarkable.

**Figure 3:**
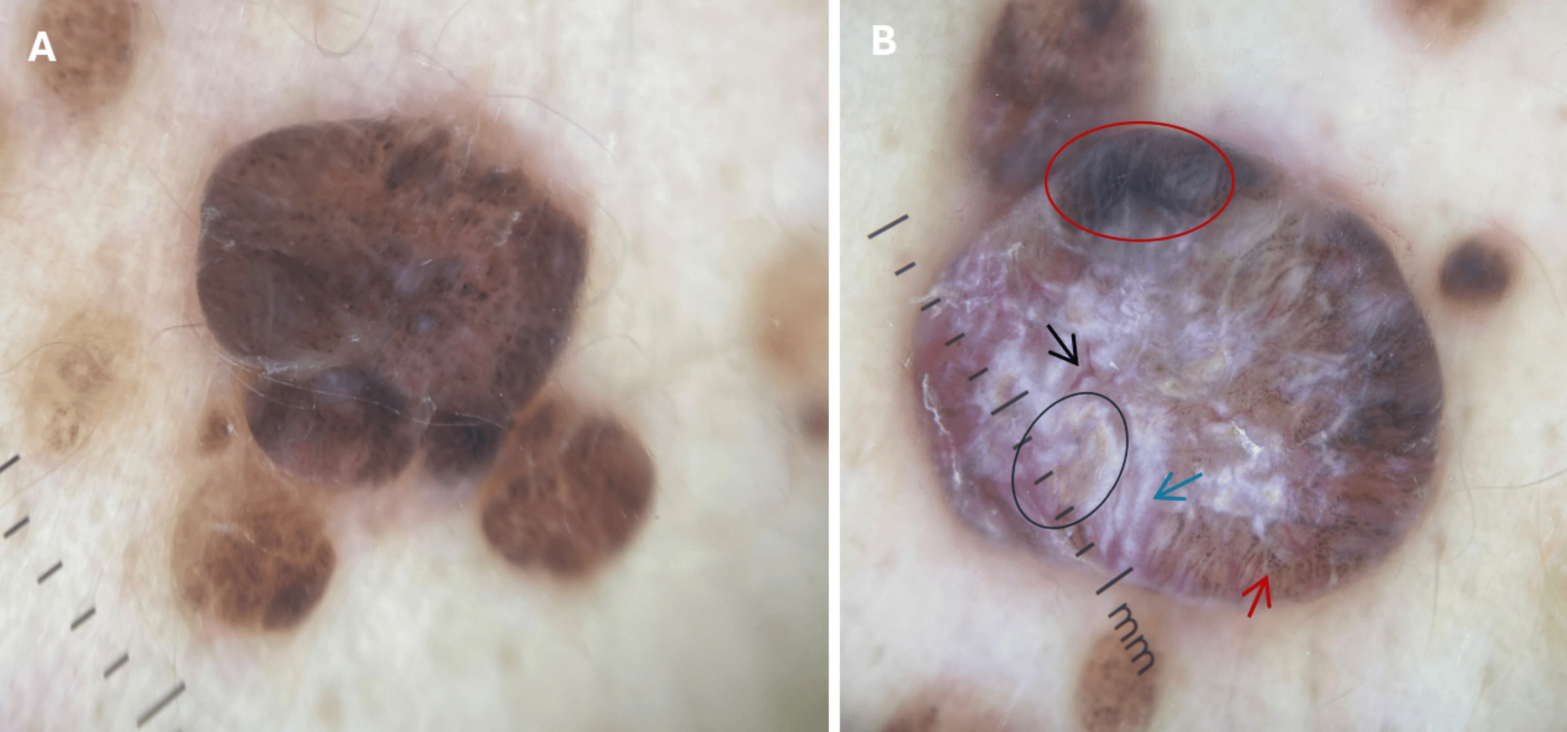
Dermoscopic examination of two axillary basal cell carcinomas. (A) Adenoid-cystic variant showing a homogeneous brown background with brown dots and globules. (B) Fibroepithelioma of Pinkus variant displaying arborizing vessels (black arrow), brown dots and globules (red arrow), crystalline structures (blue arrow), ovoid nests (red circle), and a subtle rainbow-like hue (black circle).

Dermoscopy demonstrated classic features of BCC in several lesions, including arborizing vessels, ovoid nests, and blue-gray globules. Other lesions showed atypical brown dots and globules, suggesting differential diagnoses such as adnexal tumors or seborrheic keratoses (Figure 3).

**Figure 4:**
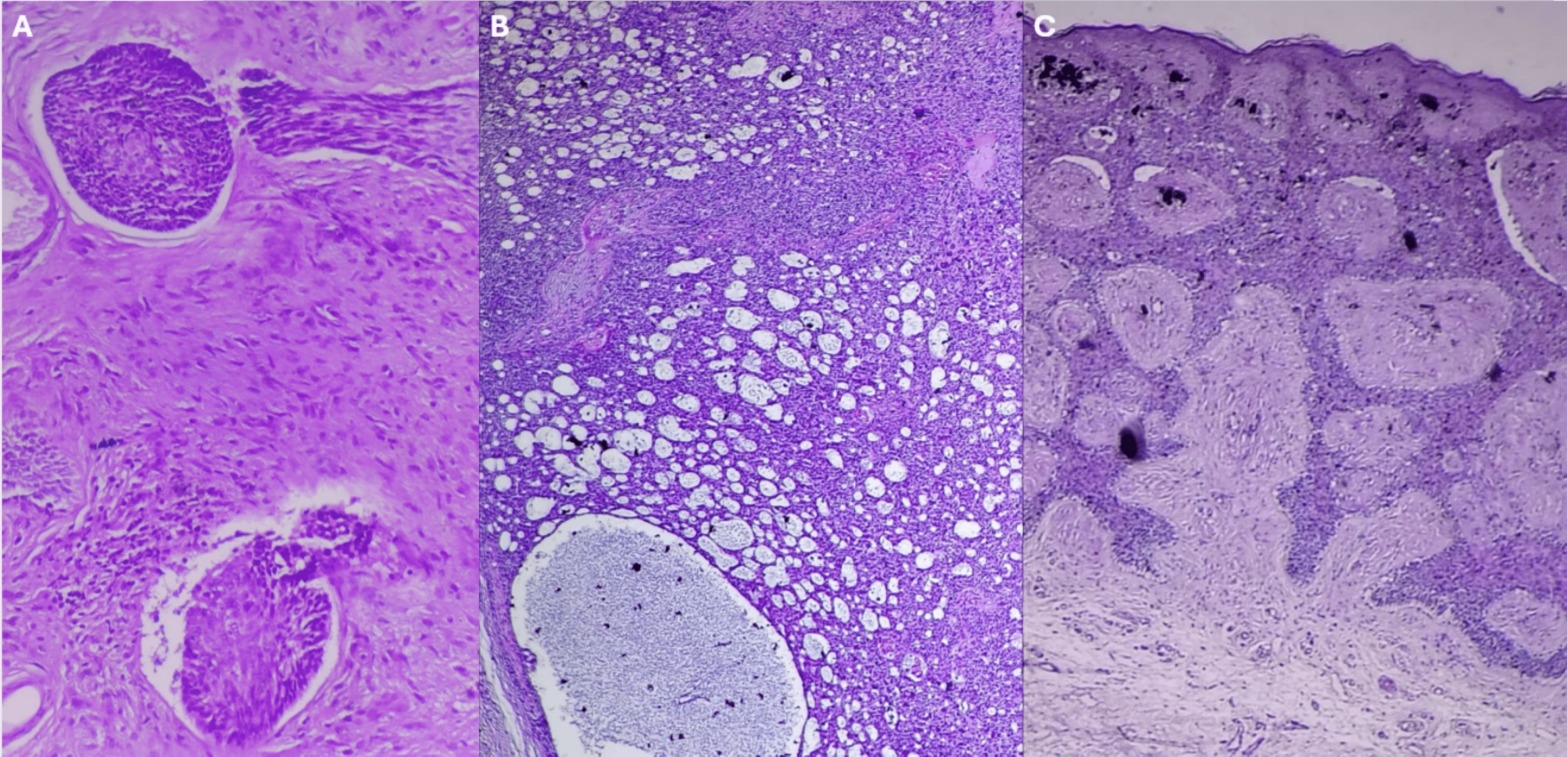
Histopathological variants of basal cell carcinoma in Gorlin-Goltz syndrome (H&E, ×100). (A) Nodular type: basaloid tumor nests with peripheral palisading and stromal retraction. (B) Adenoid-cystic type: basaloid cells arranged in a cribriform pattern with cystic spaces containing mucinous material. (C) Fibroepithelioma of Pinkus type: anastomosing epithelial strands within a fibromyxoid stroma, showing a biphasic epithelial-stromal architecture.

Multiple biopsies were performed. Histopathological examination revealed the coexistence of three distinct BCC subtypes: a nodular variant with basaloid nests and peripheral palisading; an adenoid-cystic variant characterized by gland-like structures containing mucinous material; and fibroepithelioma of Pinkus, composed of thin epithelial strands within a fibromyxoid stroma (Figure 4).

**Figure 5:**
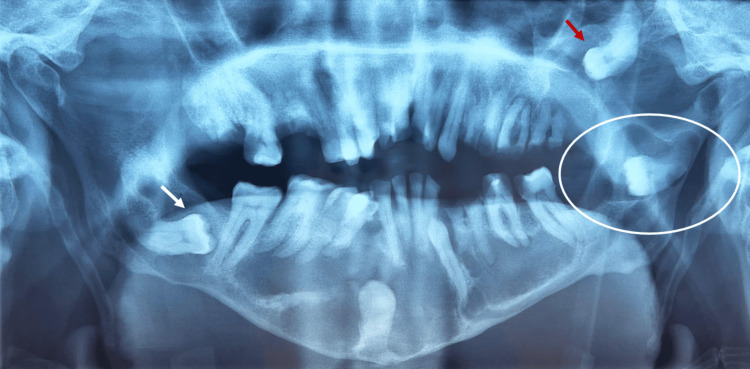
Panoramic radiograph showing an odontogenic keratocyst (white circle) associated with ectopic teeth (red arrows) and impacted teeth (white arrows).

**Figure 6:**
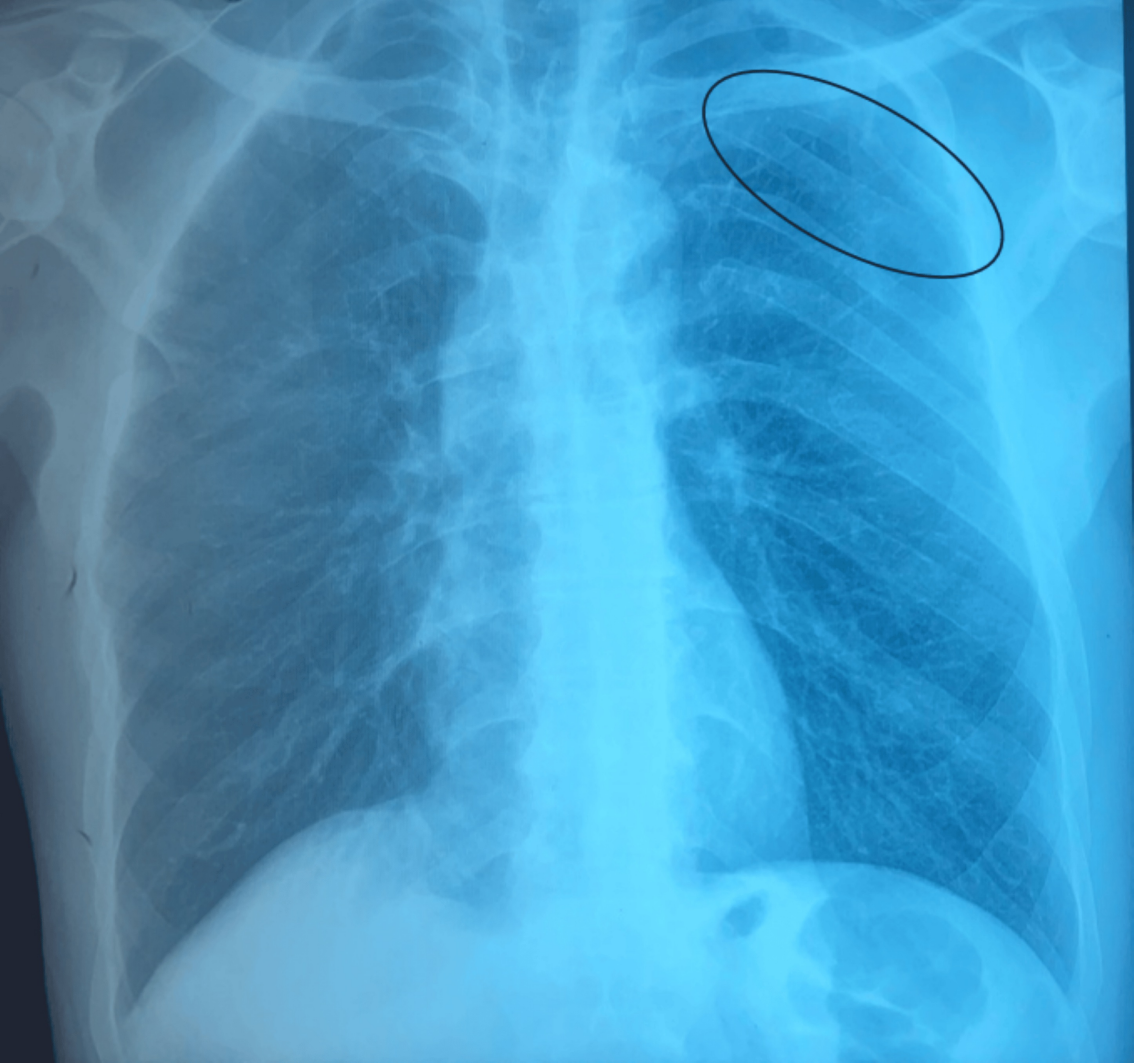
Chest radiograph revealing a bifid rib abnormality.

A panoramic dental radiograph demonstrated multiple radiolucent cystic lesions consistent with odontogenic keratocysts (Figure 5), and a standard chest radiograph revealed bifid ribs (Figure 6). Together, these findings fulfilled three major diagnostic criteria, multiple BCCs, odontogenic keratocysts, and bifid ribs, confirming the diagnosis of Gorlin-Goltz syndrome.

The patient was managed through a multidisciplinary approach involving dermatologists, plastic surgeons, maxillofacial specialists, and medical oncologists. Multiple lesions were identified, including bilateral inguinal tumors and several facial lesions. The largest ulcerated and symptomatic lesions, located in the left inguinal region, were associated with pain and bleeding and were therefore selected for surgical management. Wide local excision with approximately 5-mm safety margins was performed, and histopathological examination confirmed clear margins. Systemic treatment with the Hedgehog pathway inhibitor vismodegib, which is indicated for advanced or multiple BCCs, was discussed as a therapeutic option for controlling ongoing tumor proliferation. However, this treatment was not accessible because of its unavailability in our setting.

## Discussion

Gorlin-Goltz syndrome is caused by germline mutations in the *PTCH1* tumor suppressor gene, leading to aberrant activation of the Hedgehog signaling pathway and uncontrolled basal cell proliferation [1,2]. Its estimated prevalence ranges from 1:57,000 to 1:256,000 individuals [4]. Evans DG et al. first established major and minor diagnostic criteria for the syndrome in 1993, which were subsequently modified by Kimonis VE et al. in 1997 (Table 1) [5,6].

**Table 1:** Diagnostic criteria for Gorlin-Goltz syndrome, including major and minor criteria, and findings in the present case. BCC: Basal cell carcinoma.

Diagnostic criteria	Present case
Major criteria	
Multiple BCCs or a single BCC occurring before 20 years of age	+
Odontogenic keratocysts of the jaws, histologically confirmed	+
Three or more palmar or plantar pits	-
Bilamellar calcification of the falx cerebri	-
Bifid, fused, or markedly splayed ribs	+
First-degree relative with Gorlin-Goltz syndrome	-
Minor criteria	
Macrocephaly adjusted for height	-
Congenital abnormalities: frontal bossing, cleft lip and/or palate, moderate or severe hypertelorism, or coarse facial features	-
Other skeletal abnormalities: marked pectus deformity, syndactyly, or Sprengel deformity	-
Radiographic abnormalities: modeling defects of the hands and feet, vertebral anomalies, such as hemivertebrae, fusion or elongation of vertebral bodies, or bridging of the sella turcica	-
Ovarian fibroma	-
Medulloblastoma	-

Clinically, BCCs in Gorlin-Goltz syndrome may present as flesh-colored papules, plaques, or ulcerated tumors and can occasionally mimic benign lesions such as skin tags, nevi, hemangiomas, or adnexal tumors [4]. Although BCCs predominantly arise in photo-exposed areas, particularly the face, chest, and back, involvement of non-sun-exposed regions has also been reported [4,7]. In contrast, predominant flexural and intertriginous involvement, as observed in our patient, remains exceptionally uncommon. Our patient presented with an unusually high tumor burden exceeding 100 lesions, predominantly involving the axillary folds, inguinal folds, umbilical region, and flanks. Moreover, the occurrence of numerous BCCs in a patient with Fitzpatrick phototype IV further supports the predominant role of genetic susceptibility rather than ultraviolet-induced carcinogenesis in this syndrome.

The preferential involvement of intertriginous areas raises several hypotheses regarding the underlying pathogenic mechanisms. Chronic local factors, such as friction, maceration, and humidity, may contribute to a pro-inflammatory microenvironment within skin folds, thereby promoting tumorigenesis in genetically predisposed individuals. Although no fungal infection was documented in our patient, previous studies have shown that microorganisms such as *Candida albicans* may induce Th17 activation, leading to increased secretion of IL-17 and IL-22, cytokines found in elevated concentrations within BCC lesions [8]. These cytokines may enhance tumor cell proliferation and migration through activation of the NF-κB and STAT3 signaling pathways implicated in carcinogenesis [8,9]. Collectively, these observations support the hypothesis that local microenvironmental factors may facilitate tumor development in non-sun-exposed areas.

Another remarkable aspect of our case is the coexistence of multiple histopathological BCC subtypes in the same patient. Nodular BCC is the most frequent subtype in both sporadic and syndromic cases, whereas adenoid-cystic and fibroepithelioma of Pinkus variants are distinctly uncommon. Approximately 30% of patients with Gorlin-Goltz syndrome may exhibit two or more histological patterns simultaneously [10]. In our patient, the simultaneous presence of nodular, adenoid-cystic, and fibroepithelioma of Pinkus variants highlights marked histopathological heterogeneity and supports the concept of pluripotent differentiation of basaloid tumor cells under chronic Hedgehog pathway dysregulation [11].

From a diagnostic perspective, dermoscopy played a central role in orienting the diagnosis despite the unusual clinical presentation. Arborizing vessels, blue-gray ovoid nests, globules, and crystalline structures strongly supported the diagnosis of BCC and guided the decision to perform multiple biopsies [12].

This atypical presentation also carries important differential diagnostic implications. Intertriginous BCCs may be misdiagnosed as hidradenitis suppurativa, inverse psoriasis, fungal infections, or adnexal tumors, particularly in less-experienced clinical settings. Recognition of such unusual presentations is therefore essential to avoid diagnostic delay.

Management of Gorlin-Goltz syndrome requires a multidisciplinary approach involving dermatologists, surgeons, oncologists, maxillofacial specialists, and genetic counselors because patients may develop numerous BCCs throughout their lifetime, often requiring repeated therapeutic interventions. Conventional treatments include surgical excision, topical therapies such as imiquimod, and laser-based approaches, depending on tumor burden and lesion location [13,14]. Imiquimod has shown efficacy in selected superficial and nodular BCCs [15], while CO₂ laser resurfacing may help reduce tumor burden and improve cosmetic outcomes in patients with extensive disease [16]. More recently, targeted therapies directed against the Hedgehog signaling pathway, such as vismodegib and sonidegib, have emerged as major therapeutic advances for patients with advanced, recurrent, multiple, or surgically challenging BCCs [17,18]. In refractory advanced cases, immunotherapeutic agents such as cemiplimab may represent additional therapeutic options [19].

## Conclusions

This case highlights an unusual presentation of Gorlin-Goltz syndrome characterized by an exceptionally high burden of BCCs predominantly involving flexural and intertriginous, non-sun-exposed areas. The coexistence of multiple histopathological subtypes, including the rare fibroepithelioma of Pinkus variant, further emphasizes the marked histopathological heterogeneity associated with this syndrome. Recognition of such atypical presentations is essential to avoid diagnostic delay and ensure appropriate multidisciplinary management involving dermatologists, surgeons, oncologists, and maxillofacial specialists, as well as long-term clinical and radiological surveillance.
